# Clinical risk model to predict 28-day unplanned readmission via the accident and emergency department after discharge from acute psychiatric units for patients with psychotic spectrum disorders

**DOI:** 10.1192/bjo.2019.97

**Published:** 2020-01-28

**Authors:** Keith Hariman, Koi Man Cheng, Jenny Lam, Siu Kau Leung, Simon S. Y. Lui

**Affiliations:** Department of General Adult Psychiatry, Castle Peak Hospital, Hong Kong, China; Department of General Adult Psychiatry, Castle Peak Hospital, Hong Kong, China; Department of General Adult Psychiatry, Castle Peak Hospital, Hong Kong, China; Department of General Adult Psychiatry, Castle Peak Hospital, Hong Kong, China; Department of General Adult Psychiatry, Castle Peak Hospital, Hong Kong, China

**Keywords:** Unplanned readmission, rehospitalisation, psychotic spectrum disorders, risk model, prediction

## Abstract

**Background:**

Unplanned readmissions rates are an important indicator of the quality of care provided in a psychiatric unit. However, there is no validated risk model to predict this outcome in patients with psychotic spectrum disorders.

**Aims:**

This paper aims to establish a clinical risk prediction model to predict 28-day unplanned readmission via the accident and emergency department after discharge from acute psychiatric units for patients with psychotic spectrum disorders.

**Method:**

Adult patients with psychotic spectrum disorders discharged within a 5-year period from all psychiatric units in Hong Kong were included in this study. Information on the socioeconomic background, past medical and psychiatric history, current discharge episode and Health of the Nation Outcome Scales (HoNOS) scores were used in a logistic regression to derive the risk model and the predictive variables. The sample was randomly split into two to derive (*n* = 10 219) and validate (*n* = 10 643) the model.

**Results:**

The rate of unplanned readmission was 7.09%. The risk factors for unplanned readmission include higher number of previous admissions, comorbid substance misuse, history of violence and a score of one or more in the discharge HoNOS overactivity or aggression item. Protective factors include older age, prescribing clozapine, living with family and relatives after discharge and imposition of conditional discharge. The model had moderate discriminative power with a c-statistic of 0.705 and 0.684 on the derivation and validation data-set.

**Conclusions:**

The risk of readmission for each patient can be identified and adjustments in the treatment for those with a high risk may be implemented to prevent this undesirable outcome.

## Background

Unplanned readmission is often seen as an undesirable outcome for both the patient and the healthcare system. For the patient, being readmitted soon after discharge is disruptive to their recovery and, in the long run, is detrimental to the course of illness with poorer outcomes and may even engender dependency on psychiatric services.^1,2^ Frequent readmissions put a strain on the family, the patient's social support network and lead to social stigma.^3^ The staff may also feel demotivated over their confidence in the management of the patient.^4^ Rapid readmission, especially for patients readmitted to hospital within a month of discharge, reflects poorly on the quality of care provided by the psychiatric unit during the in-patient stay, with a lack of coordination between the discharging unit and the community care providers and may be rather costly.^5–7^ High rates of unplanned readmissions may also indicate premature discharge from the psychiatric units as hospitals in certain countries have been encouraged to shorten the patient length of stay and hospitals with high readmission rates may be penalised financially.^8^

The rate of psychiatric patients readmitted within 28 days of discharge is one of the established indicators for quality of care collected by many countries, including the USA, UK and Australia, which have set up their own benchmarking tools for comparisons across different hospitals.^9–11^ Data from the Organisation for Economic Co-operation and Development (OECD) in 2011 showed the readmission rates within 28 days of discharge for patients with schizophrenia and bipolar affective disorder, two diagnoses with higher readmission rates, were 13 and 11%, respectively.^12^ The actual figures for individual countries differ according to different research, which has reported rates up to 24.6% for patients with psychosis in the USA.^6^ However, given constraints in resources, any intervention to decrease readmission rates for these illnesses should be targeted at those with a high risk.

## Aims

This study aims to create a risk model that can predict the risk of unplanned readmissions via accident and emergency departments within 28 days of discharge from acute psychiatric units for patients with psychotic spectrum disorders, so that those with a particularly high risk and need may be identified. There has only been one risk index published to predict 30-day readmission after discharge from acute psychiatric units developed from a cohort of patients from Ontario, Canada, encompassing all psychiatric diagnosis.^13^ The model was then applied to a different group of patients in south-eastern USA with good predictive ability.^14^ Risk models are already being deployed in some countries. Scotland has deployed a risk model to calculate the risk of readmissions for all diagnoses, including psychiatric hospital readmission.^15^ However, healthcare needs and specific interventions differ for patients with different diagnoses. To our knowledge, there are no risk models published globally that target patients with psychotic spectrum disorders in particular.

## Method

### Study design and setting

This is a retrospective cohort study conducted across all eight public psychiatric units across Hong Kong that provide an in-patient treatment facility. The vast majority of the in-patient psychiatric care is provided via the public sector in Hong Kong, operated by the hospital authority. The hospital authority also operates general out-patient clinics and specialist out-patient clinics, including 11 psychiatric out-patient clinics, which share the same electronic health record platform with in-patient services. Similar to the UK, the territory of Hong Kong has been split into seven catchment areas, each with its own in-patient and out-patient services. With a population of about 7.5 million people, Hong Kong operates a heavily government-subsided healthcare system where all residents in Hong Kong are guaranteed healthcare, regardless of individual's financial capabilities. Following the global trend of deinstitutionalisation of mental healthcare, there are approximately 50 psychiatric beds per 100 000 population in Hong Kong, more than the proportion in the UK or USA but lower than the OECD average of 69 psychiatric beds per 100 000 population.^16,17^

### Data sources

The majority of the data sources for this study came from the Clinical Data Analysis and Reporting System (CDARS). CDARS compiles data from multiple online systems, including the clinical management system, the in-patient management system and the corporate drug dispensing system, dating all the way back to 1994. As all of the public healthcare facilities in Hong Kong are operated by the same healthcare provider, the system is able to pool data across every public hospital in the city. Aside from the patients’ medical records, the system is also able to retrieve administrative records relating to every single patient. Currently, CDARS is unable to retrieve and analyse free text entered into the various information systems, such as the discharge summaries, but can retrieve other categorical information. Any duplications in the data retrieval process from CDARS were cross-checked or supplemented with information from the actual free-text discharge summaries.

CDARS is also able to retrieve information from the Psychiatric Clinical Information System (PsyCIS). PsyCIS is the system that incorporates every patient's mental health history and treatment records. The system contains detailed admission and discharge records for every in-patient episode, categorical information on the patient's pertinent past history (such as any history of violence or suicide), details of the current admission and services received upon discharge. Every treating psychiatrist is also required to fill in the patient's admission and discharge Health of the Nation Outcome Scales (HoNOS).^18^

### Cohort

This study reviewed data for patients aged 18 or above and under 65 years old who were discharged from territory-wide psychiatric units over a 5-year period between 1 January 2013 and 31 December 2017. Those who have undergone an episode of unplanned readmission within 28 days of discharge were flagged by CDARS. Only patients with a primary diagnosis of psychotic spectrum disorders were included in the study, as research has suggested that the presence of psychotic experiences, even for patients whose primary diagnoses are affective disorders, is associated with worsened outcomes.^19^ Therefore, under the current ICD-10 diagnostic criteria,^20^ patients with a primary diagnosis shown in Table 1 were included.

**Table 1:** Patient diagnosis included in the study

Diagnosis	ICD-10 code
Schizophrenia, schizotypal and delusional disorders	F20–F29
Mania with psychotic symptoms	F30.2
Bipolar affective disorder, current episode manic with psychotic symptoms	F31.2
Bipolar affective disorder, current episode severe depression with psychotic symptoms	F31.5
Severe depressive episode with psychotic symptoms	F32.3
Recurrent depressive disorder, current episode severe with psychotic symptoms	F33.3

Only patients readmitted via the accident and emergency department were included, rather than through the out-patient setting, ward follow-up's or clinically admitted for procedures, where readmissions were regarded as planned.^10^ Only patients who stayed at the hospital for more than 3 days were included in the study, as the characteristics of patients who stayed for a short time differed substantially from patients with a longer length of stay and such short admissions often do not allow for enough time for the patient to be meaningfully assessed and treated.^13^ Patients with moderate or severe degrees of intellectual disability were excluded from the study as their admission may be because of behavioural problems, rather than a relapse of any comorbid psychotic spectrum disorders. For patients transferred to general hospital for acute medical conditions during their stay in a psychiatric unit and subsequently transferred back to a psychiatric unit this was counted as one episode.

### Predictor variables

The potential predictor variables selected for analysis were based on the grounds of clinical relevance, the results from the literature review and the availability of clinical data. The predictors could be broadly split into four groups of factors. The first group included data on the sociodemographic background of each patient, including age, gender, education, whether the patient's hospital admission fees were waived because they were on social welfare and where they were living after discharge. Those who were on social welfare would be exempted from any hospital charges under the hospital authority system. However, patients who have been involuntarily admitted into psychiatric units would also have their hospital admission fees waived, regardless of whether or not they receive social security. As the hospital authority system is unable to directly show whether the patient is on social security, the actual social welfare status of the patient was not used as a predictor in this study. The second group of predictors was related to the past medical and psychiatric history of the patients.

Physical comorbidities were quantified using the Charlson score, a measure of physical comorbidity that has been widely used in many different publications.^21–24^ The third group of variables was related directly to the current discharge episode, which included the legal status of the patient upon discharge, the ‘special care system (SCS)’ status of the patient and whether a conditional discharge was signed for the patient. SCS is a system that provides a framework to manage the risks and needs of patients receiving specialist psychiatric services, with input from doctors, social workers and staff from non-governmental organisations. Those who have been labelled special care or intensive care under the SCS system, the two higher levels out of a three-level system, receive more community support from other allied health practitioners, akin to the assertive outreach team in the UK, with intensive care reserved for patients with a very high degree of clinical complexity and a risk of committing serious violence.^25^ Similarly, the conditional discharge system in Hong Kong is analogous to the community treatment order in the UK, typically reserved for patients with a propensity for violence. These patients may be recalled into the specific psychiatric units should they breach certain conditions of their discharge. The last group of variables pertained to the discharge HoNOS score of the patients.

### Statistical analysis

We first examined the normality of data of the variables in our sample using Shapiro-Wilk Test. χ²-tests (for categorical data), independent *t*-tests (for parametric data) and Mann-Whitney *U*-tests (for non-parametric data) were used to detect any significant difference in each predictor between those readmitted and those who were not readmitted. Variables with more than 25% missing data were excluded from the analysis. Multivariate logistic regression models were then applied to construct a risk prediction model. To build and internally validate the predictive model, the analysis used a split-sample method, whereby the entire cohort was randomly split into two, half of which was used to derive the model whereas the other half was used to validate the model. This study used patient episodes, rather than individual patients, as the main analysis, a method commonly used by many other readmission models as well.^26^ A subsidiary analysis was also performed using patient-level data, taking only the first discharge episode within the 5-year period of a particular patient if there were more than one episode, so as to mitigate the effects of frequent admitters.

A series of four models was created by sequentially adding each group of variables mentioned above. This method was similar to the model derivation proposed by Vigod *et al*,^13^ which aimed to prioritise the variables that could be easily collected in the clinical setting requiring the least manual input while still maximising the predictive capacity of the model. The predictive model was constructed using backwards logistic regression, with the probability set at 0.05 for removal of factors. The choice of the final model was decided depending on the clinical and statistical significance of the predictors, the goodness-of-fit using the Hosmer–Lemeshow goodness-of-fit test and the model discriminative ability by the area under the receiver operating characteristic curve (AUC), also known as the c-statistic. A defined cut-off point, determined by comparing against the actual incidence of unplanned readmission, for dichotomising high risk and low risk was used to determine the sensitivity, specificity, positive predictive value (PPV) and negative predictive value (NPV) of the model. The probability for readmission for patients in the validation set was then calculated using the model from the derivation set. The final probability was then compared against the actual result of whether the patient has been readmitted or not, with another AUC computed for validation of the predictive model. All statistical analyses were carried out using Statistical Package for the Social Sciences (SPSS) software version 22.

The study has received approval from the research and ethics committees of all the study sites of this study in Hong Kong, namely New Territories West Cluster Clinical and Research Ethics Committee, Joint Chinese University of Hong Kong New Territories East Cluster Clinical Research Ethics Committee, Kowloon West Cluster Research Ethics Committee, Research Ethics Committee (Kowloon Central/Kowloon East), Institutional Review Board of the University of Hong Kong/Hospital Authority Hong Kong West Cluster and Hong Kong East Cluster Research Ethics Committee. As there was no actual patient contact in this study and only the case notes were perused, informed consent from every patient has been specifically waived. The data has been recorded in a manner that ensures that each individual could not be identified, using a non-recognisable code for each patient.

## Results

### Participant characteristics

Our sample comprised 30 707 discharge episodes, relating to 18 514 individual patients. Out of the 30 707 discharge episodes, the incidence of 28-day unplanned readmission via the accident and emergency department was 7.09%, with 1496 individual patients experiencing one or more readmission episodes. With more than 25% missing data, duration of illness was excluded in the generation of the regression models.

Table 2 describes the sociodemographic characteristics of the patients, Table 3 the past medical and psychiatric history and current discharge episode and Table 4 the HoNOS scores for the patient who were readmitted and those who were not. Taking both patient groups together, there were more women than men admitted to hospital. Most patients studied up to secondary school level and lived with their family or relatives after discharge (Table 2). Patients were diagnosed with schizophrenia, schizotypal or delusional disorders in 90.7% of the episodes, and substance misuse was the most common comorbid psychiatric illness (Table 3). Approximately 36% of patients were put on depot medications. HoNOS scores were recategorised into 0 (no problems experienced) or 1–4 (minor to severe degrees of problems experienced) as most patients were ranked 0 upon discharge (Table 4). Most factors showed significant difference between the readmitted group and the non-readmitted group (with *P* < 0.05).

**Table 2:** Socioeconomic characteristics associated with those readmitted and not readmitted

Characteristics	Readmitted (*n* = 2178)	Not readmitted (*n* = 28 529)	*P*
*Group 1: Socioeconomic background*			
Gender, *n* (%)			<0.0001*
Men	1151 (52.8)	12 945 (45.4)	
Women	1027 (47.2)	15 584 54.6	
Level of education, *n* (%)^a^			<0.0001*
Less than primary	40 (2.0)	636 (2.4)	
Primary	341 (16.7)	4791 (18.0)	
Secondary	1472 (71.9)	17 508 (65.7)	
Tertiary or above	195 (9.5)	3701 (13.9)	
Hospital fees waived because of social security, *n* (%)			<0.0001*
Yes	1036 (47.6)	8955 (31.4)	
No	1142 (52.4)	19 574 (68.6)	
Abode after discharge, *n* (%) (*n* = 23 501)^b^			<0.0001*
Alone	279 (17.4)	2511 (11.5)	
Family / relatives	823 (51.5)	14 183 (64.8)	
Institutionalised	418 (26.1)	4459 (20.4)	
No fixed abode	74 (4.6)	734 (3.4)	
Unknown	5 (0.3)	15 (0.1)	
Age (years), mean (s.d.)	40.33 (11.26)	41.89 (12.0)	<0.0001*

* *P* < 0.05.

a. Readmitted: *n* = 2048; not readmitted: *n* = 26 636.

b. Readmitted: *n* = 1599; not readmitted: *n* = 221 902.

**Table 3:** Past medical and psychiatric history and current discharge information characteristics associated with those readmitted and not readmitted

Characteristics	Readmitted (*n* = 2178)	Not readmitted (*n* = 28 529)	*P*
*Group 2: Past medical and psychiatric history*			
History of violence, *n* (%)			<0.0001*
Yes	805 (37.0)	6902 (24.2)	
No	1373 (63.0)	21 627 (75.8)	
History of suicide, *n* (%)			<0.0001*
Yes	701 (32.2)	6333 (22.2)	
No	1477 (67.8)	22 196 (77.8)	
Charlson score, median (s.d.)	0 (0.80)	0 (0.83)	<0.0001*
Number of previous admissions, median (s.d.)	5 (9.93)	2 (6.21)	<0.0001*
Duration of illness (years), mean (s.d.)	15.80 (10.84)	14.01 (11.33)	<0.0001*
*Group 3: current discharge information*			
Diagnosis, *n* (%)			0.002*
Schizophrenia (F20–F29)	2016 (92.6)	25 842 (90.6)	
Affective disorders (F30.2, F31.2, F31.5, F32.3, F33.3)	162 (7.4)	2687 (9.4)	
Comorbid substance abuse (F10–F19), *n* (%)			<0.0001*
Yes	255 (11.7)	1654 (5.8)	
No	1923 (88.3)	26 875 (94.2)	
Comorbid personality disorder (F60–F69), *n* (%)			<0.0001*
Yes	72 (3.3)	373 (1.3)	
No	2106 (96.7)	28 156 (98.7)	
Comorbid mental retardation (F70–F79), *n* (%)			<0.0001*
Yes	144 (6.6)	934 (3.3)	
No	2034 (93.4)	27 595 (96.7)	
Follow-up by clinical psychologists, *n* (%)			0.021*
Yes	710 (32.6)	9996 (35.0)	
No	1468 (67.4)	18 533 (65.0)	
Follow-up by occupational therapists, *n* (%)			<0.0001*
Yes	144 (6.6)	934 (3.3)	
No	2034 (93.4)	27 595 (96.7)	
Follow-up by medical social workers, *n* (%)			0.165
Yes	601 (27.6)	8271 (29.0)	
No	1577 (72.4)	20 258 (71.0)	
Follow-up by community psychiatric service, *n* (%)			0.093
Yes	976 (44.8)	13 316 (46.7)	
No	1202 (55.2)	15 213 (53.3)	
Discharged against medical advice, *n* (%)			0.767
Yes	36 (1.7)	530 (1.9)	
No	2142 (98.3)	27 999 (98.1)	
Legal status upon discharge, *n* (%)			<0.0001*
Involuntary	168 (7.7)	3211 (11.2)	
Voluntary/informal	2010 (92.3)	25 318 (88.7)	
Conditional discharge, *n* (%)			0.070
Yes	33 (1.5)	595 (2.1)	
No	2145 (98.5)	27 934 (97.9)	
Special care system status, *n* (%)^a^			<0.0001*
Conventional care	1551 (71.2)	23 018 (80.9)	
Special care	585 (26.9)	4999 (17.6)	
Intensive care	42 (1.9)	442 (1.6)	
Clozapine prescribed, *n* (%)			0.143
Yes	271 (12.4)	3254 (11.4)	
No	1907 (87.6)	25 275 (88.6)	
Depot prescribed, *n* (%)			0.010*
Yes	833 (38.2)	10 126 (35.5)	
No	1345 (61.8)	18 403 (64.5)	
Length of stay (days), mean (s.d.)	58.51 (223)	69.32 (216.46)	<0.0001*

* *P* < 0.05.

a. Not readmitted: *n* = 28 459.

**Table 4:** Health of the Nation Outcome Scales (HoNOS) scores associated with those readmitted and not readmitted

	Readmitted (*n* = 1911)		Not readmitted (*n* = 25 726)		*P*
	*n*	%	*n*	%	
*Group 4: HoNOS scores*					
HoNOS item 1: overactive, aggressive, disruptive or agitated					0.014*
Score 0	1829	95.7	24 890	96.8	
Score 1–4	82	4.3	836	3.2	
HoNOS item 2: non-accidental self-injury					0.309
Score 0	1896	99.2	25 462	99.0	
Score 1–4	15	0.8	264	1.0	
HoNOS item 3: problem drinking or drug taking					<0.0001*
Score 0	1778	93.0	24 897	96.8	
Score 1–4	133	7.0	829	3.2	
HoNOS item 4: cognitive problems					0.013*
Score 0	1782	93.2	24 335	94.6	
Score 1–4	129	6.8	1391	5.4	
HoNOS item 5: physical illness or disability problems					0.122
Score 0	1780	93.1	23 710	92.2	
Score 1–4	131	6.9	2016	7.8	
HoNOS item 6: problems with hallucinations and delusions					0.557
Score 0	1576	82.5	21 351	83.0	
Score 1–4	335	17.5	4375	17.0	
HoNOS item 7: problems with depressed mood					0.930
Score 0	1860	97.3	25 048	97.4	
Score 1–4	51	2.7	678	2.6	
HoNOS item 8: other mental and behavioural problems					0.406
Score 0	1829	95.7	24 515	95.3	
Score 1–4	82	4.3	1211	4.7	
HoNOS item 9: problems with relationships					<0.0001*
Score 0	1626	85.1	22 607	87.9	
Score 1–4	285	14.9	3119	12.1	
HoNOS item 10: problems with activities of daily living					0.181
Score 0	1781	93.2	24 171	94.0	
Score 1–4	130	6.8	1555	6.0	
HoNOS item 11: problems with living conditions					0.026*
Score 0	1809	94.7	24 629	95.7	
Score 1–4	102	5.3	1097	4.3	
HoNOS item 12: problems with occupation and activities					0.809
Score 0	1632	85.4	22 022	85.6	
Score 1–4	279	14.6	3704	14.4	

* *P* < 0.05.

### Derivation of the risk prediction model and measuring the model's accuracy

Owing to missing data, the final number of episodes used to derive the logistic regression was 10 219. The results of each regression model are presented in Table 5. The *P-*values for the Hosmer–Lemeshaw tests of all four models were above 0.05, meaning that the fitted models were satisfactory and the observed and predicted values of the dependent variables were different. The addition of more groups of variables resulted in higher c-statistics, indicating a logistic regression model with more predictive variables resulted in better model discriminative power. As a result, model 4 was chosen as the final model for the prediction of unplanned readmissions. The model had a c-statistic of 0.705, indicating a moderate discriminative power. Taking a cut-off point of 8% as the risk of readmission, similar to the actual readmission risk of 7.09% during the study period, the model achieved a sensitivity of 47.2%, specificity of 79.3%, PPV of 14.8% and a NPV of 95.2%. When the model was applied to the validation set, which consisted of the other half of the total episodes (*n* = 10 643), the c-statistic was 0.684, again indicating a moderate discriminative power.

**Table 5:** Results of the Hosmer–Lemeshow test and discriminative power of the various models

Model and predictors	*P-*value of Hosmer–Lemeshow test	AUC/c-statistic
Model 1, predictors: group 1	0.097	0.619
Model 2, predictors: groups 1–2	0.444	0.688
Model 3, predictors: groups 1–3	0.374	0.699
Model 4, predictors: groups 1–4	0.078	0.705

AUC, area under the receiver operating characteristic curve; HoNOS, Health of the Nation Outcome Scales.

Table 6 shows the predictive variables that were statistically significant in model 4, the final prediction model. All predictors achieved statistical significance with a *P*-value of less than 0.05 apart from certain variables in the abode after discharge. Patients with more admissions in the past or who had comorbid substance use disorders were more likely to be readmitted. Those with a history of violence or who exhibited signs of agitation or aggressiveness upon discharge, as signified by a score of 1 or more in the first item of HoNOS, were also more likely to be readmitted within 28 days. A prescription of clozapine, older age or the imposition of conditional discharge status prior to discharge conferred a protective effect. Those who were discharged to live with their family or relatives were less likely to be readmitted than those who lived alone.

**Table 6:** Predictors of unplanned readmission within 28 days of discharge

Predictor	Odds ratio	95% CI		*P*
		Lower	Upper	
Number of previous admissions	1.064	1.055	1.072	<0.0001*
Comorbid substance misuse	1.494	1.163	1.918	0.002*
History of violence	1.295	1.085	1.546	0.004*
HoNOS item 1: overactive, aggressive, disruptive or agitated	1.496	1.057	2.116	0.023*
Conditional discharge	0.285	0.132	0.614	0.001*
Clozapine prescribed	0.703	0.546	0.906	0.007*
Age	0.979	0.972	0.986	<0.0001*
Abode after discharge				
Alone	Reference	–	–	–
Family/relatives	0.609	0.488	0.761	<0.0001*
Institutionalised	0.829	0.646	1.064	0.140
No fixed abode	0.289	0.510	1.222	0.789
Unknown	0.647	0.156	19.837	1.761

* *P* < 0.05.

### Subsidiary analysis

In the subsidiary analysis using patient-level data, there were 18 514 patients included in the analysis. Half of these patients was used to derive the prediction model. As with the episode-level data, there were significant amounts of missing data. A total of 5548 and 6936 patients were included in the final derivation model and validation model. Higher number of previous admissions, comorbid substance misuse and history of violence were once again found to be the risk factors for unplanned readmission along with no fixed abode after discharge, whereas older age and living with family or relatives provided a protective effect. Even though conditional discharge was included in the model, the variable did not achieve statistical significance. The model had a *P*-value of 0.836 on the Hosmer–Lemeshaw test, indicating a model with satisfactory fit. The c-statistic of the derivation and validation model was 0.658 and 0.661, respectively, both showing moderate discriminative power.

## Discussion

### Main findings

This study has derived and internally validated a risk model using a split-sample approach to predict unplanned readmissions within 28 days of discharge from psychiatric units via the accident and emergency department for patients with psychotic spectrum disorders; this is the first model published in the literature to predict the risk of readmission for this particular group of patients. The c-statistics of the derivation (0.705) and validation (0.684) set both indicated that the model was of moderate discriminative power, on a par with other prediction models for readmissions, including those for other medical conditions and higher than the model used to predict readmissions for all psychiatric diagnoses, where the c-statistic was 0.631 and 0.630 for the derivation and validation sets.^13,26,27^

Like many other predictive models, the model derived in this current study has a moderate sensitivity (47.2%), low PPV (14.8%) but a relatively high specificity (79.3%) and NPV (95.2%) using a cut-off point of 8% as the risk of readmission. The moderate sensitivity means that only half of the patients subsequently readmitted were identified by the model, although this may be improved by adjusting the cut-off point. The relatively low incidence of unplanned readmissions imposed a ceiling on the PPV and many of the patients with a positive test were eventually not readmitted. This combination of moderate sensitivity, low PPV and high NPV is similar to prediction models for suicide and violence, demonstrating that risk predictions are generally difficult in mental health given the multifactorial associations.^28,29^

Given the findings from this study, there are certain interventions that psychiatrists may deliver to their patients, particularly those with a high risk, to decrease the rate of unplanned readmissions, such as psychoeducation, medication education and reconciliation and coping skills training that have been proven to be effective.^30–32^ Psychiatrists should also place more emphasis on the treatment of comorbid substance use disorders and find ways to manage any residual aggression or overactivity upon discharge, two risk factors found to be significant in this study. Other effective measures noted in the literature, such as post-discharge telephone follow-up and efforts to ensure timely follow-up, may be provided by other allied health professionals or mental health hotlines.^30^ These interventions have a low cost and low likelihood of adverse events and can be delivered to many of those predicted with a high risk of readmission. Even if some of patients were falsely predicted to be readmitted, because of the low PPV, it is unlikely that they would be harmed with these practices and those who tested negative would not receive unnecessary interventions given the high NPV.^33^

### Interpretation of our findings and comparison with findings from other studies

Apart from the large sample size used to derive the prediction model, another strength of this study is the inclusion of both pre-admission and treatment information in the prediction retrieved from an objective clinical population-level database. If unplanned readmissions are used as an indicator for the quality of care provided, then clinicians should be able to alter their practice in a meaningful way to decrease the occurrence of the event.^34^ Models that only incorporate historical data and ignore information related to the current episode may not be able to suggest any clinically and statistically significant interventions that can decrease unplanned readmissions. The research published by Vigod *et al* on readmissions for all psychiatric diagnoses incorporated certain parameters related to the index hospital admission, such as the diagnosis, length of stay, threat to self or others, planned discharge and self-care ability, but omitted other treatment factors and left out the results from the rating scales on the patient's symptomatology.^13^ The predictor variables are also readily retrievable and the risk model may therefore be applied or validated in other countries.

The results from this study show that prescribing clozapine and establishing a conditional discharge requirement on the patient may be protective and decrease the risk of unplanned readmissions. A large trial comparing the effectiveness of antipsychotic treatments for patients with schizophrenia has already proven that clozapine is associated with the lowest rates of treatment failure, as defined by many parameters including psychiatric hospital readmission.^35^ Other research conducted in the UK found that patients with schizophrenia or schizoaffective disorder who have been newly prescribed with clozapine have a decreased risk of readmission, even if these patients had more severe psychopathology and poorer functional status.^36^ A systematic review on the impact of clozapine on hospital use also found that clozapine reduced the proportion of people admitted to hospital compared with controls.^37^ The authors hypothesised that the reason for this protective effect may be because of the need for ongoing regular haematological monitoring, where deteriorations in mental state may be detected earlier. Clozapine is also known to reduce aggressive behaviour in patients, independent of its antipsychotic effect.^38^

This study has also shown the protective effect of conditional discharge orders. It is possible that patients with this order may have received more robust assessments before discharge and be more adherent with their treatment and follow-up attendance, thereby reducing the risk of readmission. However, a Cochrane review on compulsory community and involuntary out-patient treatment for people with severe mental disorders found that there was no clear statistical difference in readmissions to hospital by 12 months.^39^

The protective effect of older age or living with family or relatives in reducing readmissions has been described in some literature, although other studies have often showed mixed results.^8,40^ Similarly, the increased risk of readmission associated with a history of violence, residual symptoms of overactivity or agitation as measured by HoNOS item 1 and comorbid substance misuse has been described in some literature, although these findings were often not found in other studies.^8,41,42^ The only consistent variable that has a proven predictive effect is a higher number of previous admissions, sometimes over a pre-defined period in the past, for example 3 years.^26,43,44^ Interestingly, one piece of research found that interventions such as symptom education and service continuity, previously noted to be effective to prevent readmissions, may become statistically insignificant for those with four or fewer previous admissions, suggesting that the benefits may be dependent on the pattern of use of healthcare services.^31^

The length of stay of the patient was not found to be significant in this study, even though some studies have showed that a shorter length of stay during the index hospital admission led to a higher risk of readmission.^3,41,45^ This has become an important finding as many countries have pressed for a shorter length of stay and some psychiatrists may discharge a patient prematurely before recovery in an attempt to hasten the patient's reintegration into society in the community.^46^ This may be because of the relatively long length of stay of patients in Hong Kong compared with other countries, with less pressure for early discharge, and data being partly skewed by the discharge episodes of certain patients who have lived in psychiatric institutions for years.^41^

Depot medications were also not found to be significant in preventing readmission, contrary to the findings of nationwide cohort studies or publications solely comparing the risk of readmission to hospital for patients taking oral antipsychotics versus depot medications.^35,47^ However, other papers have found that depot medications had no association with the risk of readmission.^14,48^ Depot medications are sometimes prescribed for patients with a high risk of poor drug adherence, which some have suggested to be the cause for relapse. However, some pieces of research have failed to find an association, suggesting that medication non-adherence is more likely to be associated with delayed readmissions to psychiatric units, rather than rapid readmissions such as the time frame of 28 days used in this study.^6,45,49^

Although there is a statistically significant result on the χ²-test for the effect of gender and readmission, logistic regression has failed to demonstrate gender as a significant predictor of readmission. The female predominance of discharges is similar to the overall statistics in Hong Kong across all diagnoses, possibly because women are more likely to seek help for their medical problems with better insight and exhibit more bizarre behaviour and affective symptoms upon presentation, thus leading to admission to hospital.^50–52^

### Comparison of patient-level and episode-level data results

Our subsidiary analysis using patient-level data rather than episode-level data revealed that the predictors of unplanned readmission included comorbid substance misuse, history of violence, no fixed abode after discharge and higher number of previous admissions, whereas older age and living with family or relatives were protective factors. A history of violence, HoNOS item 1, prescription of clozapine and conditional discharge were not statistically significant factors in the predictive model. There are several possible reasons why the predictors were different for the patient- and episode-level data. First, the sample size used for regression analysis for the patient-level data was about half that of the episode-level data. A smaller sample size might not have enough power to detect any statistically significant results. In the episode-level data analysis, the highest odds ratio for any single predictor was less than 1.5. Researchers have also found that the effects of several predictors changed over time, so what was once not significant may become important factors in subsequent episodes.^41^ This is exemplified with HoNOS item 1, prescription of clozapine and conditional discharge not found to be statistically significant factors in the patient-level data. However, the finding of these three as significant results in the episode-level data sheds light on areas that may be potentially amenable and worthy of attention, particularly if the patient has any residual aggression or overactivity upon discharge, given that some other predictors are unmodifiable in nature. Finally, it is rather difficult to engage patients who have no fixed abode after discharge and it can be more difficult to deliver any transition arrangements or community interventions to them.

### Limitations and future directions

This study focused on readmissions via the accident and emergency department, where there is a higher chance that the readmissions would be unplanned. On the other hand, readmissions may occur via out-patient clinics and may also be unplanned, though this would be hard to delineate in practice, as noted by the OECD data.^12^ This would therefore affect the sample of patients identified in the readmitted group. The data used in this study was obtained through CDARS, which is more limited in scope compared with a review of discharge summaries or patient interviews. The reliance on psychiatrist input has contributed to the vast amounts of missing data and different definitions of certain data points, such as the duration of illness.

Unlike other studies, this study has not incorporated any standardised tests into the prediction model simply because such instruments are not routinely used to assess the patients upon discharge, instead relying solely on HoNOS scores. HoNOS scores crudely reflect the patient's psychotic symptoms in its measurement and have been argued to exhibit only moderate interrater reliability in some literature.^53–55^ Furthermore, there have not been any studies on the validity and reliability of the use of HoNOS in Hong Kong and the duration of training on the use of HoNOS across the city is not unified, meaning that the contribution of HoNOS item 1 as a predictor variable should be further investigated. Other studies have tried to incorporate other rating instruments in their prediction model for unplanned readmission; however, inclusion did not improve the power of the model.^13,44^ In addition, the model has not adequately assessed the post-discharge social and health system variables for their contribution to readmissions. Data on the out-patient treatment of the patient, such as the time to follow-up, was not incorporated in this study, which may in turn affect readmission rates.^56^ It is also likely that the differing practices of the various hospitals and attending psychiatrists will have affected the decision to admit a patient to hospital, thereby influencing the readmission rates.^57^

There is therefore much work to be done in the future to look into other predictor variables, including the incorporation of social and system factors. One possible method would be the use of artificial intelligence and deep learning. Much of the free-text data in the discharge summaries were left out in the analysis. The backwards regression model also had its inherent flaws in removing predictors one by one, without considering how the dropped factors might interact with each other when other variables were dropped, and models with large sample sizes can easily drive the *P*-values of variables to be less than 0.05.^58^ There has already been research published that has used deep learning methods to predict certain health outcomes, such as in-hospital mortality, by scanning through various electronic health record data and converting the information into data that could be analysed, achieving discriminative powers of up to 0.93–0.94 in the c-statistic, although the 30-day unplanned readmission model could only reach a c-statistic of 0.75–0.76.^59^ Research that focused on predicting violence and suicide also noted that the discriminative power was vastly improved when machine learning approaches were adopted.^60,61^

### Implications

Despite the debate on whether readmissions should be used as a proxy for the quality of care provided, many hospitals are still routinely assessed on readmission rates and are penalised if the rates are too high. The actual reasons why patients are rapidly readmitted may be influenced by a variety of patient-levels factors, broader social and environmental factors such as social support, and hospital- and health-system-level factors. As all of the predictors in this model can be easily generated without much manual effort, the system can calculate the risk of readmission for each patient. This automated work process is particularly important as it would require no additional clinician manipulation to calculate the risk of each patient and a concerted approach may then be adopted from prior to discharge transitioning to out-patient care.

This clinical risk model is the first study to predict unplanned readmissions within 28 days for patients with psychotic spectrum disorders. Both the derivation and validation model were able to achieve moderate discriminative ability, with a c-statistic of 0.705 and 0.684, respectively. With this predictive model, the risk of readmission for each patient can be calculated and those predicted to be at high risk may be identified. Adjustments can then be made to their discharge arrangements or treatment may be implemented, focusing on the risk factors noted in this study and other effective measures mentioned in the literature, to prevent this undesirable outcome.

## Data Availability

The data related to this study is currently being stored at a safe location in accordance with Hospital Authority's policy on medical records.

## References

[1] Evans LJ, Harris V, Newman L, Beck A. Rapid and frequent psychiatric readmissions: associated factors. Int J Psychiatry Clin Pract 2017; 21: 271–6. 28554237 10.1080/13651501.2017.1324037

[2] Frick U, Frick H, Langguth B, Landgrebe M, Hübner-Liebermann B, Hajak G. The revolving door phenomenon revisited: time to readmission in 17,415 patients with 37,697 hospitalisations at a German psychiatric hospital. PLoS One 2013; 8: e75612. 24116059 10.1371/journal.pone.0075612 PMC3792950

[3] Kalseth J, Lassemo E, Wahlbeck K, Haaramo P, Magnussen J. Psychiatric readmissions and their association with environmental and health system characteristics: a systematic review of the literature. BMC Psychiatry 2016; 16: 376–84. 27821155 10.1186/s12888-016-1099-8 PMC5100223

[4] Lien L. Are readmission rates influenced by how psychiatric services are organized? Nord J Psychiatry 2002; 56: 23–8. 11869461 10.1080/08039480252803873

[5] Callaly T, Trauer T, Hyland M, Coombs T, Berk M. An examination of risk factors for readmission to acute adult mental health services within 28 days of discharge in the Australian setting. Australas Psychiatry 2011; 19: 221–5. 21682619 10.3109/10398562.2011.561845

[6] Lorine K, Goenjian H, Kim S, Steinberg AM, Schmidt K, Goenjian AK. Risk factors associated with psychiatric readmission. J Nerv Ment Dis 2015; 203: 425–30. 25974053 10.1097/NMD.0000000000000305

[7] Hamilton JE, Rhoades H, Galvez J, Allen M, Green C, Aller M, Factors differentially associated with early readmission at a university teaching psychiatric hospital. J Eval Clin Pract 2015; 21: 572–8. 25756751 10.1111/jep.12335

[8] Donisi V, Tedeschi F, Wahlbeck K, Haaramo P, Amaddeo F. Pre-discharge factors predicting readmissions of psychiatric patients: a systematic review of the literature. BMC Psychiatry 2016; 16: 1–17. 27986079 10.1186/s12888-016-1114-0 PMC5162092

[9] NHS Digital. *Clinical Indicators Team. NHS Outcomes Framework* NHS Digital, 2019 (https://indicators.hscic.gov.uk/webview/).

[10] Australian Mental Health Outcomes and Classification Network. *National Mental Health Benchmarking Project Manual Part 3: Technical specifications for the national KPIs* National Mental Health Strategy version 1.1, 2006.

[11] Health Services Advisory Group. *Thirty-Day All-Cause Unplanned Readmission following Psychiatric Hospitalization in an Inpatient Psychiatric Facility (IPF)* Centers for Medicare & Medicaid Services Measures Inventory Tool, 2019.

[12] OECD. Unplanned hospital re-admissions for patients with mental disorders In Health at a Glance 2013: OECD Indicators: 120–1. OECD Publishing, 2013.

[13] Vigod SN, Kurdyak PA, Seitz D, Herrmann N, Fung K, Lin E, READMIT: A clinical risk index to predict 30-day readmission after discharge from acute psychiatric units. J Psychiatr Res 2015; 61: 205–13. 25537450 10.1016/j.jpsychires.2014.12.003

[14] Roque AP, Findlay LJ, Okoli C, El-Mallakh P. Patient characteristics associated with inpatient psychiatric re-admissions and the utility of the READMIT Clinical Risk Index. Issues Ment Health Nurs 2017; 38: 411–9. 28448224 10.1080/01612840.2016.1269856

[15] Health and Social Care Programme Scotland. A Report on the Development of SPARRA Version 3 - Developing Risk Prediction to Support Preventive and Anticipatory Care in Scotland. NHS National Services Scotland, 2012.

[16] Organisation for Economic Cooperation and Development. *OECD Health Statistics 2017: Health Care Resources.* OECD, 2018.

[17] Hospital Authority Statistics and Workforce Planning Department. *Inpatient Bed Occupancy Rate* Hospital Authority, Hong Kong, 2019.

[18] Wing JK, Beevor AS, Curtis RH, Park SGB, Hadden J, Burns A. Health of the Nation Outcome Scales (HoNOS). Br J Psychiatry 1998; 172: 11–8. 9534825 10.1192/bjp.172.1.11

[19] Van Os J, Reininghaus U. Psychosis as a transdiagnostic and extended phenotype in the general population. World Psychiatry 2016; 15: 118–24. 27265696 10.1002/wps.20310 PMC4911787

[20] World Health Organization. The ICD-10 Classification of Mental and Behavioural Disorders: Clinical Descriptions and Diagnostic Guidelines. World Health Organization, 1992.

[21] Romano PS, Roos LL, Jollis JG. Adapting a clinical comorbidity index for use with ICD-9-CM administrative data: differing perspectives. J Clin Epidemiol 1993; 46: 1075–9. 8410092 10.1016/0895-4356(93)90103-8

[22] Boaz TL, Becker MA, Andel R, Van Dorn RA, Choi J, Sikirica M. Risk factors for early readmission to acute care for persons with schizophrenia taking antipsychotic medications. Psychiatr Serv 2013; 64: 1225–9. 23945797 10.1176/appi.ps.003382012

[23] Šprah L, Dernovšek MZ, Wahlbeck K, Haaramo P. Psychiatric readmissions and their association with physical comorbidity: a systematic literature review. BMC Psychiatry 2017; 7: 2. 10.1186/s12888-016-1172-3 PMC5210297 28049441

[24] Barker LC, Gruneir A, Fung K, Herrmann N, Kurdyak P, Lin E, Predicting psychiatric readmission: sex-specific models to predict 30-day readmission following acute psychiatric hospitalization. Soc Psychiatry Psychiatr Epidemiol 2018; 53: 139–49. 29124290 10.1007/s00127-017-1450-5

[25] Task Group on Special Care System Review. Operations Manual on Special Care System for Psychiatric Patients. Hospital Authority, Hong Kong, 2017.

[26] Zhou H, Della PR, Roberts P, Goh L, Dhaliwal SS. Utility of models to predict 28-day or 30-day unplanned hospital readmissions: an updated systematic review. BMJ Open 2016; 6: e011060. 10.1136/bmjopen-2016-011060 PMC4932323 27354072

[27] Kansagara D, Englander H, Salanitro A, Kagen D, Theobald C, Freeman M, Risk prediction models for hospital readmission: a systematic review. JAMA 2011; 306: 1688–98. 22009101 10.1001/jama.2011.1515 PMC3603349

[28] Fazel S, Singh JP, Doll H, Grann M. Use of risk assessment instruments to predict violence and antisocial behaviour in 73 samples involving 24 827 people: systematic review and meta-analysis. BMJ 2012; 345: e4692. 22833604 10.1136/bmj.e4692 PMC3404183

[29] Belsher BE, Smolenski DJ, Pruitt LD, Bush NE, Beech EH, Workman DE, Prediction models for suicide attempts and deaths: a systematic review and simulation. JAMA Psychiatry 2019; 76: 642–51. 30865249 10.1001/jamapsychiatry.2019.0174

[30] Vigod SN, Kurdyak PA, Dennis CL, Leszcz T, Taylor VH, Blumberger DM, Transitional interventions to reduce early psychiatric readmissions in adults: systematic review. Br J Psychiatry 2013; 202: 187–94. 23457182 10.1192/bjp.bp.112.115030

[31] Prince JD. Practices preventing rehospitalization of individuals with schizophrenia. J Nerv Ment Dis 2006; 194: 397–403. 16772855 10.1097/01.nmd.0000222407.31613.5d

[32] Schmidt-Kraepelin C, Janssen B, Gaebel W. Prevention of rehospitalization in schizophrenia: results of an integrated care project in Germany. Eur Arch Psychiatry Clin Neurosci 2009; 259: S205–212. 19876680 10.1007/s00406-009-0056-7

[33] Carter G, Milner A, McGill K, Pirkis J, Kapur N, Spittal MJ. Predicting suicidal behaviours using clinical instruments: systematic review and meta-analysis of positive predictive values for risk scales. Br J Psychiatry 2017; 210: 387–95. 28302700 10.1192/bjp.bp.116.182717

[34] Durbin J, Lin E, Layne C, Teed M. Is readmission a valid indicator of the quality of inpatient psychiatric care? J Behav Heal Serv Res 2007; 34: 137–50. 10.1007/s11414-007-9055-5 17437186

[35] Tiihonen J, Mittendorfer-Rutz E, Majak M, Mehtälä J, Hoti F, Jedenius E, Real-world effectiveness of antipsychotic treatments in a nationwide cohort of 29823 patients with schizophrenia. JAMA Psychiatry 2017; 74: 686–93. 28593216 10.1001/jamapsychiatry.2017.1322 PMC5710250

[36] Kesserwani J, Kadra G, Downs J, Shetty H, MacCabe JH, Taylor D, Risk of readmission in patients with schizophrenia and schizoaffective disorder newly prescribed clozapine. J Psychopharmacol 2019; 33: 449–58. 30616489 10.1177/0269881118817387 PMC6431783

[37] Land R, Siskind D, McArdle P, Kisely S, Winckel K, Hollingworth SA. The impact of clozapine on hospital use: a systematic review and meta-analysis. Acta Psychiatr Scand 2017; 135: 296–309. 28155220 10.1111/acps.12700

[38] Meyer JM, Stahl SM. The Clozapine Handbook. Cambridge University Press, 2019.

[39] Kisely SR, Campbell LA, O'Reilly R. Compulsory community and involuntary outpatient treatment for people with severe mental disorders. Cochrane Database Syst Rev 2017; 3: CD004408. 28303578 10.1002/14651858.CD004408.pub5 PMC6464695

[40] Sfetcu R, Musat S, Haaramo P, Ciutan M, Scintee G, Vladescu C, Overview of post-discharge predictors for psychiatric re-hospitalisations: a systematic review of the literature. BMC Psychiatry 2017; 17: 227–41. 28646857 10.1186/s12888-017-1386-z PMC5483311

[41] Tulloch AD, David AS, Thornicroft G. Exploring the predictors of early readmission to psychiatric hospital. Epidemiol Psychiatr Sci 2016; 25: 181–93. 25703270 10.1017/S2045796015000128 PMC6998497

[42] Burke RE, Donzé J, Schnipper JL. Contribution of psychiatric illness and substance abuse to 30-day readmission risk. J Hosp Med 2013; 8: 450–5. 23589474 10.1002/jhm.2044

[43] Donisi V, Tedeschi F, Salazzari D, Amaddeo F. Pre- and post-discharge factors influencing early readmission to acute psychiatric wards: Implications for quality-of-care indicators in psychiatry. Gen Hosp Psychiatry 2016; 39: 53–8. 26804775 10.1016/j.genhosppsych.2015.10.009

[44] Moss J, Li A, Tobin J, Weinstein IS, Harimoto T, Lanctôt KL. Predictors of readmission to a psychiatry inpatient unit. Compr Psychiatry 2014; 55: 426–30. 24405773 10.1016/j.comppsych.2013.11.019

[45] Hamilton JE, Passos IC, De Azevedo Cardoso T, Jansen K, Allen M, Begley CE, Predictors of psychiatric readmission among patients with bipolar disorder at an academic safety-net hospital. Aust N Z J Psychiatry 2016; 50: 584–93. 26377747 10.1177/0004867415605171

[46] Rylander M, Colon-Sanchez D, Keniston A, Hamalian G, Lozano A, Nussbaum AM. Risk factors for readmission on an adult inpatient psychiatric unit. Qual Manag Health Care 2016; 25: 22–31. 26783864 10.1097/QMH.0000000000000077

[47] MacEwan JP, Kamat SA, Duffy RA, Seabury S, Chou JW, Legacy SN, Hospital readmission rates among patients with schizophrenia treated with long-acting injectables or oral antipsychotics. Psychiatr Serv 2016; 67: 1183–8. 27417897 10.1176/appi.ps.201500455

[48] Hung Y-Y, Chan H-Y, Pan Y-J. Risk factors for readmission in schizophrenia patients following involuntary admission. PLoS One 2017; 12: e0186768. 29073180 10.1371/journal.pone.0186768 PMC5658080

[49] Craig TJ, Fennig S, Tanenberg-Karant M, Bromet EJ. Rapid versus delayed readmission in first-admission psychosis: quality indicators for managed care? Ann Clin Psychiatry 2000; 12: 233–8. 11140925 10.1023/a:1009038627449

[50] Barajas A, Ochoa S, Obiols JE, Lalucat-Jo L. Gender differences in individuals at high-risk of psychosis: a comprehensive literature review. ScientificWorldJournal 2015; 2015: 430735. 25685840 10.1155/2015/430735 PMC4312997

[51] Ochoa S, Usall J, Cobo J, Labad X, Kulkarni J. Gender differences in schizophrenia and first-episode psychosis: a comprehensive literature review. Schizophr Res Treatment 2012; 2012: 916198. 22966451 10.1155/2012/916198 PMC3420456

[52] Chang WC, Tang JYM, Hui CLM, Chiu CPY, Lam MML, Wong GHY, Gender differences in patients presenting with first-episode psychosis in Hong Kong: a three-year follow up study. Aust N Z J Psychiatry 2011; 45: 199–205. 21261552 10.3109/00048674.2010.547841

[53] Adams M, Palmer A, O'Brien JT, Crook W. Health of the nation outcome scales for psychiatry: are they valid? J Ment Heal 2000; 9: 193–8.

[54] Pirkis JE, Burgess PM, Kirk PK, Dodson S, Coombs TJ, Williamson MK. A review of the psychometric properties of the Health of the Nation Outcome Scales (HoNOS) family of measures. Health Qual Life Outcomes 2005; 3: 76. 16313678 10.1186/1477-7525-3-76 PMC1315350

[55] Brooks R. The reliability and validity of the Health of the Nation Outcome Scales: validation in relation to patient derived measures. Aust New Zeal J Psychiatry 2000; 34: 504–11. 10.1080/j.1440-1614.2000.00755.x 10881976

[56] Marcus SC, Chuang C-C, Ng-Mak DS, Olfson M. Outpatient follow-up care and risk of hospital readmission in schizophrenia and bipolar disorder. Psychiatr Serv 2017; 68: 1239–46. 28669289 10.1176/appi.ps.201600498

[57] Moss J, Nauranga D, Kim D, Rosen M, Wang K, Lanctot K. Variation in admission rates between psychiatrists on call in a university teaching hospital. Ann Gen Psychiatry 2018; 17: 30. 30008791 10.1186/s12991-018-0199-x PMC6040071

[58] Urach C, Zauner G, Wahlbeck K, Haaramo P, Popper N. Statistical methods and modelling techniques for analysing hospital readmission of discharged psychiatric patients: A systematic literature review. BMC Psychiatry 2016; 16: 413–21. 27863514 10.1186/s12888-016-1128-7 PMC5116202

[59] Rajkomar A, Oren E, Chen K, Dai AM, Hajaj N, Liu PJ, Scalable and accurate deep learning for electronic health records. NPJ Digit Med 2018; 1: 18. 31304302 10.1038/s41746-018-0029-1 PMC6550175

[60] Walsh CG, Ribeiro JD, Franklin JC. Predicting risk of suicide attempts over time through machine learning. Clin Psychol Sci 2017; 5: 457–69.

[61] Menger V, Spruit M, van Est R, Nap E, Scheepers F. Machine learning approach to inpatient violence risk assessment using routinely collected clinical notes in electronic health records. JAMA Netw Open 2019; 2: e196709. 31268542 10.1001/jamanetworkopen.2019.6709 PMC6613290

